# Brussels Declaration: a vehicle for the advancement of tobacco and alcohol industry interests at the science/policy interface?

**DOI:** 10.1136/tobaccocontrol-2018-054264

**Published:** 2018-06-25

**Authors:** Jim McCambridge, Mike Daube, Martin McKee

**Affiliations:** 1 Department of Health Sciences, University of York, York, UK; 2 Faculty of Health Sciences, Curtin University, Perth, Western Australia, Australia; 3 ECOHOST, London School of Hygiene and Tropical Medicine, London, UK

**Keywords:** tobacco industry, surveillance and monitoring, public policy, harm reduction

## Abstract

The case for policies to be based on evidence appeared to gain a major boost with the publication of the Brussels Declaration, apparently with support from many leading scientists and institutions. Yet, as we show in this analysis, there are major concerns about how it was developed and, in particular, the extensive involvement of tobacco and alcohol industry actors. We describe how its coverage of conflicts of interest and vested interests is consistent with the perspectives of these same actors. The process of developing the Declaration successfully involved science advisors, other senior officials in governments and politicians in its preparation. Despite this, the final Declaration fails to address the need for safeguards to protect the integrity of science or policy from corporate interests, including in relation to the tobacco industry. This undermines Article 5.3 of the Framework Convention on Tobacco Control which seeks to protect public health policies from interference by the tobacco companies. More broadly, the Declaration offers potential to serve as a vehicle for advancing the vested interests of corporate sectors in public policymaking and appears to have been regarded in this way by a range of organisations related to the alcohol industry. This exercise is now being extended to the continent of Africa, which is strategically important to both the tobacco and alcohol industries. It will be important to study carefully to what extent initiatives like this form part of the global political strategies of tobacco and alcohol industry actors.

## Introduction

The Brussels Declaration is a statement of ethics and principles for science and society policymaking.^1^ This arose from discussions at the prestigious World Science Forum, and was designed to attract attention. It was launched formally at the American Association for the Advancement of Science in February 2017, accompanied by an announcement in the journal *Nature*.^2^ It raises questions about the integrity of scientists and calls on them to be less ‘aloof and perhaps less arrogant’.^1^ It calls on policymakers to be more accountable and, crucially, demands that voices of interest groups are heard in the policy debate. At a time when facts are increasingly being questioned in some political fora, it has the potential to be very influential.

Its avowed goal, evidence-based policymaking, will be widely shared (see box 1 for summary text). Yet while it makes much of the need for research integrity and transparency, the Declaration fails to disclose its own origins and funding, or the interests of those involved. Moreover, on closer inspection there are many curious aspects to the organisation of what purports to be a ‘bottom up’ initiative.^1^ This analysis examines the background to the initiative and process of developing it, before drawing attention to concerns about the detailed content and prospects for influence, and finally addressing some of the issues raised.

Box 1 **Brussels Declaration summary text^1^**

**Science and policy—a crucial relationship**

1. Science is a fundamental pillar of knowledge-based societies.
2. Science can help provide the evidence base for public policy.
3. Sound public policy is crucial for the direction and priorities of science.
4. The dialogue between science and policy is never straightforward.

**What we expect from the scientific community**

1. The integrity of science needs to be clear and the integrity of scientists providing advice must be unimpeachable.
2. The full range of scientific disciplines should be included; notably, the social sciences can play a key role in improving how the public may react or adapt.
3. Scientists must learn to use established communication channels for providing policy advice more effectively and be less aloof and perhaps less arrogant.
4. Scientists must listen and respond to criticism.

**What we expect from the policymaking community**

1. Policymakers must listen, consult and be held accountable.
2. Ethical consideration of the impact of policy decisions is crucial
3. Policymakers have to challenge science to deliver on public investment.
4. Policymakers should be willing to justify decisions, particularly where they deviate from independent scientific advice.
5. Policy makers should acknowledge the potential for bias and vested interests contrary to the scientific consensus

**What we expect from the public, media, industry and interest groups**

1. The public plays a critical role in influencing policy and must be included in the decision-making process.
2. Industry is an investor in knowledge generation and science and has every right to have its voice heard.
3. Interest groups similarly have every right to have their voice heard as guardians of the common good or legitimate sectoral interests.
4. Advice from any source to policymaking must acknowledge possible bias.

**What needs to change: how scientific advice and greater inclusivity need to be integrated more effectively**

1. Scientific advice must be more involved in all stages of the policymaking process.
2. Policymaking must learn to cope with the speed of scientific development and include greater foresight and policy anticipation.
3. Societal investment in science will always require priority setting; nevertheless, advances in public health deserve special attention.

## A process that permits tobacco company involvement

The initiative is reported to have originated with a communications consultancy called ‘Sci-Com’ (http://www.sci-com.eu/main/index.php/about-us/who-we-are) which is ‘dedicated to delivering solutions to communicating the most challenging science’. From the outset, published documentation on the Sci-Com website has given prominence to ‘harm reduction’ and addiction to tobacco, alcohol and drugs.^3^ We can find no declarations of interest in any of its publications or reports.

The Declaration states that it was developed following a series of consultations with more than 300 stakeholders from 2012 onwards.^2^ One hundred and sixty-five are named in the report, 62 of whom, including the organisers, were present at the concluding event in 2016, and another 103 who attended earlier consultation events or contributed text.^1^ There is little information available on how participants were selected, or who was involved, apart from those named. The Declaration lists those participating, including distinguished public health scientists, science journalists, senior science advisors and other officials in governments, current and former members of parliaments and government ministers, and representatives of industry. This is not surprising; there are many fora where these groups come together to discuss science policy.

What is surprising, however, is the substantial presence of representatives from the tobacco industry and, especially, British American Tobacco (BAT). Indeed, with participation by its Group Scientific Director, Chief Scientific Officer, Head of Biosciences at Group R & D, International Scientific Affairs Manager, as well as the Chief Medical Officer, Head of R & D and International Public and Scientific Affairs Director of its wholly owned Nicoventures,^1^ BAT’s representation exceeds that of any other company. There are also two representatives each from the American Chamber of Commerce to the European Union and the European Smokeless Tobacco Council (BAT is a member of both, as are other tobacco companies), and from Swedish Match^1^ (which is also a member of the latter).

This was very different from the first meeting in 2012, from which the tobacco industry was excluded as a condition for the participation of the European Commission’s health directorate, DG Sanco.^3^ This was in compliance with Article 5.3 of the Framework Convention on Tobacco Control (FCTC)^4^ which precludes the tobacco industry and related vested interests from having any input into public health policy. We see no indications that the various named individuals linked to the European Commission were involved after the first meeting.

It is unclear why the situation changed subsequently, with BAT personnel becoming prominently involved, for example, through multiple authorships among 20 ‘thought leader’ essays.^1^ The content of the Declaration expects rather a lot of scientists and policymakers, and rather less of industry actors; it questions the integrity of scientists, calls on them to be less ‘aloof’ and demands accountability for their use of public funding. It also calls on policymakers to be more accountable. It proposes that they give much greater access to industry, the organisations that represent its interests and ‘the public’, which although unsaid is likely to include many ‘grassroots organisations’ created by industry, sometimes described as ‘astroturf’,^5^ as well as those who advocate on their behalf on social media, whether real people or ‘bots’.^6^

In this context, it is worth considering further the ‘we’ whose voices make this declaration.

Richard Horton, the editor of the *Lancet*, who attended the first event and was unaware of any subsequent process involving tobacco companies, was quoted, as if as offering an endorsement, despite not having seen, or been aware of, the Declaration.^7^ This calls into question the process described to produce this statement of principles and recommendations, particularly as it declares that: ‘All too often, who those experts are, how they are chosen and how reliable their advice really is, is open to question.’^1^ We asked the founder and chief executive officer of Sci-Com by email whether they were; ‘willing to share details of all those who funded the work that led to the Declaration, and also advise whether you have any policy on taking funding from the tobacco industry.’ We received no reply. In these circumstances, a careful analysis of the wording of the Declaration seems justified.

## Detailed content analysis

The Declaration advocates an alternative to ‘a continued dangerous slide into the realm of policy-biased evidence,’^1^ though what this alternative is or might be is unclear. The content is broad in scope, covering a wide range of issues at the science/policy interface. For example, there is much content on science advice, science policy and public health, and importantly in relation to prospects for future influence, the initiative secured the involvement of high-level scientific advisors to public institutions, as well as other senior officials.

The view that ‘vested interests can be beneficial,’^1^ as long as they are declared has long been advocated by the tobacco industry, notwithstanding evidence of studies being designed to give the intended results, including the creation of doubt,^8^ and by meta-analyses showing that the funding source can be an important factor in the results obtained.^9–12^ Scientists are encouraged not to discount ‘citizen science’, despite extensive evidence of how corporations have supported social media campaigns to challenge accepted scientific wisdom^13^ and used front groups to shift the discourse and attack legitimate researchers.^14^ The tobacco industry’s long history of distorting scientific evidence means that many leading medical journals will not consider for publication any material resulting from tobacco industry funding.^15 16^ The challenging issues inevitably raised by any tobacco company involvement in any consideration of the relationships between science and policy are entirely ignored.

The Declaration champions views promulgated by organisations such as the Institute of Economic Affairs,^17^ an organisation endorsed by some British politicians,^18^ which is among the lowest scoring on an index of funding transparency^19^ but which is known to have received tobacco industry funding. It has a long history of opposing health organisations and measures they propose to reduce the harms of tobacco and alcohol.^17^ It regards public funding of science as a source of bias in policymaking,^20^ as part of its long-standing hostility to public institutions, deploying this argument to attack what is described in the Declaration as the ‘scientific-political establishment’.^1^ It seems unconcerned by any risk of bias from funding of research by those with vested commercial interests.

The scale on which tobacco companies are still seeking to undermine tobacco control measures has been recently documented by the WHO,^21^ and confirmed yet again by media exposés such as the acquisition, by Reuters, of a large repository of industry documents^22^ and a series of reports in the *Guardian* newspaper.^23^ The policies fiercely opposed by tobacco companies are crucial for achieving the Sustainable Development Goals and other global targets to reduce non-communicable diseases.^24^ Yet the Declaration fails to mention the FCTC or Article 5.3.^4^ On the contrary, although not identifying the tobacco industry as such, it states that: ‘Industry is not to be shunned when it comes to policy-making’^1^ and ‘industry should speak out more when denied access to important policy-making.’^1^

By promoting industry involvement in policymaking without any explicit safeguards for the management of vested interests, the Declaration is bound to raise concerns. These are exacerbated by its treatment of commercial conflicts of interest, which it portrays as straightforward to manage, compared with the non-financial conflicts of interest of scientists: ‘*industry is too often perceived as suffering from fatal conflicts of interest and its views are therefore dismissed. In fact, commercial conflicts of interests are fairly easy to deal with if they are properly declared…Ideological, personal or academic conflicts of interest, on the other hand, are much harder to detect or deal with.*’^1^ This ignores both the vast body of evidence about the difficulties in managing commercial conflicts, as well as the obligation on academics to declare any conflicts of interest, while implying, without evidence, that some participants in policy debates from the health arena base their concerns on ‘ideological, personal or academic conflicts’.^1^

Many other statements in the Brussels Declaration seem entirely reasonable (see box 1). Yet there are also some that, especially if they are taken in isolation, such as those discussed here, give considerable cause for concern.

## The nexus of tobacco and alcohol industry strategies?

The content of the Declaration is reminiscent of the promotion of ‘sound science’ and ‘good epidemiology’ practice in previous decades, by tobacco companies such as Phillip Morris.^25^ In that instance, perfectly reasonable propositions were articulated, alongside the subtle inclusion of content that served key purposes for the company.^25^ For example, it was proposed that any findings where the relative risk was less than two could be discounted because of the risk of unrecorded confounding, which conveniently eliminated most of the evidence on the harms of passive smoking. This earlier initiative was successful in achieving the support of key scientific organisations such as the International Epidemiological Association, which was unaware of the involvement of Phillip Morris.^25^ There are also similarities with an approach previously used by BAT in relation to ‘better regulation’; promoted to enable the inclusion of mandatory consultation with all stakeholders affected by policy decision-making, including those otherwise excluded from policymaking.^26^

It is noteworthy that tobacco industry actors would not have been permitted to be involved overtly in discussions of tobacco-specific scientific or policy issues in line with Article 5.3 of the FCTC.^4^ This means that broader focused initiatives such as the Brussels Declaration, and the examples discussed above, may be strategically important to tobacco companies. The specific tactics used to advance tobacco industry interests in science and/or policy warrant careful examination; for example, a National Dialogue on Cancer in the USA, was later shown to have been orchestrated by the tobacco industry.^27^

While we have focused so far on the tobacco industry, there are also seven different alcohol industry organisations named.^1^ Although there are, for example, pharmaceutical actors involved including four from Johnson and Johnson, no other corporate sector matches the tobacco and alcohol industries in extent of involvement, perhaps because of the chosen prominence of addiction and public health issues. Subtle reformulations of key research and policy ideas are also characteristic of alcohol industry actors,^28^ though we know far less about their methods than we do about tobacco industry actors, due to lack of access to internal company documents and the underdevelopment of research using other data sources.

The involvement of the Brewers of Europe is, however, noteworthy. In 2006 that organisation convened a group of researchers who concluded that: ‘violence is a subjective term which is fairly nebulous and elastic,’ which could undermine research on alcohol-related violence.^29^ In 2014 Spirits Europe, another alcohol industry trade association involved, criticised individual scientists, research projects and research agendas that it regarded as biased, proposing an alternative public health research agenda.^30^ The Portman Group has previously paid large sums to researchers who wrote anonymous critiques of a WHO sponsored alcohol policy evidence review.^31^ The Scotch Whisky Association led the campaign to delay the Scottish Government’s policy on minimum unit pricing^32^ despite compelling evidence^33^ in its favour.

Further, there are similarities with earlier alcohol industry approaches, notably the 1997 Dublin ‘Principles of Cooperation Among the Beverage Alcohol Industry, Governments, Scientific Researchers, and the Public Health Community’ that sought to promote partnerships between the research community and industry actors.^34^ This initiative was sponsored by the International Centre for Alcohol Policies (ICAP), a body presented as a reliable and authoritative source of public health and policy guidance, but formed and funded by global alcohol producer companies, and in part by Phillip Morris through their ownership for decades of the Miller Brewing Company.^35^ (The successor organisation to ICAP, the International Alliance for Responsible Drinking now claims the ‘Dublin Principles’ as their own.^36^) The Dublin statement drew the attention of other tobacco companies, most notably BAT. The most senior BAT figure involved in the Brussels Declaration said of the Dublin Principles in 2000 in an internal company email to addressees including another senior figure named in the Brussels Declaration that: ‘They make interesting reading. Something to aspire to?’^35 37^ Thus, BAT has a long-standing interest in the Brussels Declaration type of initiative and, despite much rhetorical distancing from tobacco companies by alcohol industry actors, it is clear that they are willing to work closely together on influencing the science/policy interface.^38^

We identified 20 of 165 names listed in the Declaration as directly representing tobacco or alcohol industry organisations; others also have such associations. For example, a Nicoventures-funded scientific paper on tobacco harm reduction was authored with a company employee involved in the Declaration^39^ and another individual’s companies have previously consulted for BAT.^40^

## And on to Africa

As smoking rates in traditional markets decline, low-income countries, including those in Africa, are seen as important growth areas for tobacco companies, while increasing disposable incomes make these countries attractive to other global sectors, including alcohol. The Brussels Declaration process was used as the model for a further initiative convened by Sci-Com; in June 2017 participants from nine African countries met in Cape Town with some of the same ‘thought leaders’ from five other countries ‘to put an African-specific process on the rails’.^41^ This Sci-Com initiative again broadly addressed many aspects of the relationships between science and policy, this time in the context of Africa, and again recruited senior scientific figures. Similarly, there was a prominent focus on ‘harm reduction’ for tobacco and alcohol, and although there was no BAT presence, among those involved were the Head of Communications for sub-Saharan Africa for Phillip Morris International, and a senior alcohol industry trade association representative, as well as political, pharmaceutical company, and governmental figures.^41^ A 4-year process leading to a further declaration statement on ethics and principles of science and policy is intended. It seeks to address similar issues to those covered in the Brussels Declaration in an African context.^41^

## Issues and implications

A range of issues are raised by the Brussels Declaration. We emphasise five key points. First there are questions to be answered by Sci-Com about the Brussels Declaration process, including how participants were selected, who actually attended the 2013–2015 events, how much involvement the named participants had in the final Declaration and how the costs were met. Similar questions should be asked about the African extension to this project.

Second, there are issues relating to how to protect from vested interests those networked forms of governance, in which consultancies, and other proliferating non-traditional organisations such as think tanks^42^ are participants. In particular, the exclusion of tobacco companies from involvement in public health policymaking required by Article 5.3 of the FCTC may be undermined by their activities in broader domains of policymaking. Tobacco companies are adept at subtle steering of others’ actions, including putting their own arguments in the voices of others.^43^ Consequently, there is a well-founded need for monitoring of their activities to incorporate attention to third parties, in order to prevent the subversion of public health policies.^21^ Consideration should be given to extending the governance model of Article 5.3 to identify those in receipt of any tobacco company funding for any purpose, notwithstanding the complex pathways through which industry funding may be channelled. Although there is no equivalent international treaty for alcohol, similar issues have been raised about the alcohol industry,^44 45^ particularly as its representatives are extensively involved in alcohol policymaking^38^ and there is no governance model operating to address these concerns. If evidence is to inform policymaking, there must be full transparency about funding and other conflicts of interest from all involved to protect against distortions of the evidence. Norms concerning the integrity of the process need to be articulated clearly, in the public interest, and there is a clear need to build on what has been learnt from research on the tobacco industry to see how it can be applied to other sectors, increasing knowledge about what has been termed the corporate determinants of health.^46^

Third, there is a question of how to manage potential direct or indirect interactions with tobacco company personnel and others acting on their behalf. While governmental officials should be cognisant of their duties to avoid tobacco company actors in respect of public health, vigilance is also warranted in relation to science and other areas of public policy. Scientists and policymakers accepting invitations to meetings need to undertake due diligence about the credentials of the organisers and ensure that their names and reputations will not be inappropriately used. It is also important that scientific and health-related organisations take care to avoid other ways in which their names and reputations can later be associated inadvertently with industry-supported initiatives, such as the commercial hire of meeting rooms.^47^

Fourth, the Brussels Declaration is far from the only recent development in which tobacco and alcohol industry actors have been operating in the research community on science and policy. The recent announcement of a substantial investment by Phillip Morris International in a ‘Foundation for a Smoke-Free World’^48^ has been widely criticised by leading scientists and health authorities, including in this journal,^49^ and by the WHO, which noted: ‘that research and advocacy funded by tobacco companies and their front groups cannot be accepted at face value.’^50^ Such activities require dedicated in-depth studies of whether the claims made are consistent with the activities of the actors involved,^51^ and the development of new research agendas. From using research funding to bias the evidence base as a principal strategy in earlier decades, to subtle alterations to scientific norms, it now appears that science policy and the institutional governance of research have been identified by tobacco and alcohol industry actors as new targets for their attention in the contexts of developing global political strategies.

The final issue centres on the uses to which the Brussels Declaration may be put, and more broadly, how vested interests may use this and other scientific issues to influence policy.^52 53^ It is premature to assess the extent to which this Declaration has been used successfully as an instrument of influence. We do already know that it has been used to gain access to key figures in policymaking on public health, science and universities in the Brussels Declaration, and now the subsequent African process. We cannot at this stage assess whether it has influenced policymakers’ views on conflicts of interest or on the evaluation of scientific evidence, or whether there are any discernible impacts on public health or science policies. However, it does seem likely that it can be used in this way by vested interests. It is thus of particular concern that the continent of Africa, which is clearly of major strategic importance to the tobacco and alcohol industries,^21 54–56^ is now being targeted.

## Conclusion

The Brussels Declaration argues for the need to protect science from distortion by vested interests. Yet it appears to be a vehicle for advancing the vested interests of certain corporate sectors. Calls for research integrity reflect core values of the research community. They should not be used as instruments to undermine science or to assist harmful industries. It will be important to study carefully to what extent this initiative, and others like it, do form part of the global political strategies of tobacco and alcohol industry actors, and the extent to which these are successful in influencing public health and science policies, in order to counter any adverse effects on population health.

**What this paper adds**

- The Brussels Declaration claims to promote evidence-based policy but a lack of transparency, and the extent of involvement of tobacco and alcohol industry actors, raise questions about whether it is all that it seems.
- The detailed content of the Brussels Declaration contains material on conflicts of interest and vested interests that is conducive to the furtherance of corporate interests in science and public health policymaking.
- Despite the involvement of leading scientists, government science advisors and other senior officials, the Declaration lacks the safeguards needed to protect the integrity of science or policy from corporate interests, including in relation to the tobacco and alcohol industries.
- Prominent scientists and opinion leaders need to be alert to ways in which they may inadvertently be used to promote harmful industry agendas, and there is a need for careful study of how, and how far, initiatives such as this influence public health and science policies.

## References

[1] Sci-com. Ethics and principles for science and society policy making 20 point global plan: The Brussels declaration. 2017 http://www.sci-com.eu/main/docs/Brussels-Declaration.pdf

[2] Kazatchkine M, Kinderlerer J, Gilligan A Brussels declaration: twenty-point plan for science policy. Nature 2017;541:289 10.1038/541289a 28102242

[3] Sci-com. Science in the public interest volume 1: harm reduction. 2013 http://www.sci-com.eu/main/docs/scicom-science-in-the-public-interest-vol1.pdf

[4] World Health Organization. Technical resource on the protection of public health policies with respect to tobacco control from commercial and other vested interests of the tobacco industry for country implementation of WHO Framework Convention on Tobacco Control Article 5.3. Geneva, 2012.

[5] Lyon TP, Maxwell JW Astroturf: interest group lobbying and corporate strategy. *J Econ Manage Strategy* 2004;13:561–97. 10.1111/j.1430-9134.2004.00023.x

[6] van der Tempel J, Noormohamed A, Schwartz R, et al Vape, quit, tweet? Electronic cigarettes and smoking cessation on Twitter. Int J Public Health 2016;61:249–56. 10.1007/s00038-016-0791-2 26841895

[7] Horton R Offline: difficult truths about a post-truth world. Lancet 2017;389:1282 10.1016/S0140-6736(17)30878-4 28379142

[8] Diethelm PA, Rielle JC, McKee M The whole truth and nothing but the truth? The research that Philip Morris did not want you to see. Lancet 2005;366:86–92. 10.1016/S0140-6736(05)66474-4 15993237

[9] Scollo M, Lal A, Hyland A, et al Review of the quality of studies on the economic effects of smoke-free policies on the hospitality industry. Tob Control 2003;12:13–20. 10.1136/tc.12.1.13 12612356 PMC1759095

[10] Perlis RH, Perlis CS, Wu Y, et al Industry sponsorship and financial conflict of interest in the reporting of clinical trials in psychiatry. Am J Psychiatry 2005;162:1957–60. 10.1176/appi.ajp.162.10.1957 16199844

[11] Cristea IA, Gentili C, Pietrini P, et al Sponsorship bias in the comparative efficacy of psychotherapy and pharmacotherapy for adult depression: meta-analysis. Br J Psychiatry 2017;210:16–23. 10.1192/bjp.bp.115.179275 27810891

[12] Lundh A, Lexchin J, Mintzes B, et al Industry sponsorship and research outcome. Cochrane Database Syst Rev 2017;2:Mr000033 10.1002/14651858.MR000033.pub3 28207928 PMC8132492

[13] Hager N Dirty politics: how attack politics is poisoning New Zealand’s political environment. Wellington: Craig Potton Publishing, 2014.

[14] Apollonio DE, Bero LA The creation of industry front groups: the tobacco industry and "get government off our back". Am J Public Health 2007;97:419–27. 10.2105/AJPH.2005.081117 17267719 PMC1805008

[15] Godlee F, Malone R, Timmis A, et al Journal policy on research funded by the tobacco industry. BMJ 2013;347:f5193 10.1136/bmj.f5193 24129479

[16] The Tobacco Control Senior Editorial Team (listed in alphabetical order): Joaquin Barnoya JC, Coral Gartner, Lisa Henriksen, Sarah Hill, Ruth E, Malone, Richard O’Connor. Why tobacco control still won’t publish tobacco industry funded work, even if the funding is laundered through PMI’s new ‘independent’ foundation. 2017 http://blogs.bmj.com/tc/2017/10/23/why-tobacco-control-still-wont-publish-tobacco-industry-funded-work-even-if-the-funding-is-laundered-through-pmis-new-independent-foundation/

[17] Tobacco Tactics. Institute of economic affairs. 2016 http://www.tobaccotactics.org/index.php?title=Institute_of_Economic_Affairs

[18] Institute for Economic Affairs. IEA at 60 – Rt Hon Sajid Javid MP 19 June 2015. 2015 https://iea.org.uk/multimedia/video/iea-at-60-rt-hon-sajid-javid-mp

[19] Who funds you? The UK campaign for think tank transparency. 2017 http://whofundsyou.org/org/institute-of-economic-affairs

[20] Smith KE, Collin J, Hawkins B, et al The pursuit of ignorance. BMJ 2016;352:i1446 10.1136/bmj.i1446 26979784 PMC4843966

[21] World Health Organization. WHO Report on the global tobacco epidemic 2017: Monitoring tobacco use and prevention policies Geneva. 2017 http://www.who.int/tobacco/global_report/en/

[22] Reuters. The Philip Morris files. 2017 https://www.reuters.com/investigates/section/pmi/

[23] The Guardian. The Guardian view on big tobacco: stop the spread: Editorial. 2017 https://www.theguardian.com/commentisfree/2017/jul/12/the-guardian-view-on-big-tobacco-stop-the-spread

[24] GBD 2016 SDG Collaborators. Measuring progress and projecting attainment on the basis of past trends of the health-related sustainable development goals in 188 countries: an analysis from the global burden of disease study 2016. Lancet 2017;390:1423–59. 10.1016/S0140-6736(17)32336-X 28916366 PMC5603800

[25] Ong EK, Glantz SA Constructing "sound science" and "good epidemiology": tobacco, lawyers, and public relations firms. Am J Public Health 2001;91:1749–57. 10.2105/AJPH.91.11.1749 11684593 PMC1446868

[26] Smith KE, Fooks G, Gilmore AB, et al Corporate coalitions and policy making in the European Union: how and why British American Tobacco promoted "Better Regulation". J Health Polit Policy Law 2015;40:325–72. 10.1215/03616878-2882231 25646389 PMC4668595

[27] Goldberg P How PR firms created “dialogue” structure used by cancer groups and tobacco clients. 2017 https://cancerletter.com/articles/20171006_2/

[28] McCambridge J, Kypri K, Drummond C, et al Alcohol harm reduction: corporate capture of a key concept. PLoS Med 2014;11:e1001767 10.1371/journal.pmed.1001767 25490717 PMC4260782

[29] McKee M A European alcohol strategy. BMJ 2006;333:871–2. 10.1136/bmj.39003.629606.BE 17047004 PMC1626340

[30] Spirits Europe. The body of evidence: policy-making and Research. 2014 http://spirits.eu/files/34/cp.as-050-2014-the-body-of-evidence.pdf

[31] Smith R Questioning academic integrity. BMJ 1994;309:1597–8. 10.1136/bmj.309.6969.1597 7819927 PMC2541970

[32] Holden C, Hawkins B ‘Whisky gloss’: the alcohol industry, devolution and policy communities in Scotland. Public Policy Adm 2013;28:253–73. 10.1177/0952076712452290

[33] Meier PS, Holmes J, Angus C, et al Estimated effects of different alcohol taxation and price policies on health inequalities: a mathematical modelling study. PLoS Med 2016;13:e1001963 10.1371/journal.pmed.1001963 26905063 PMC4764336

[34] Stenius K, Babor TF The alcohol industry and public interest science. Addiction 2010;105:191–8. 10.1111/j.1360-0443.2009.02688.x 19804465

[35] Jernigan DH Global alcohol producers, science, and policy: the case of the International Center for Alcohol Policies. Am J Public Health 2012;102:80–9. 10.2105/AJPH.2011.300269 22095330 PMC3490544

[36] IARD General Principles for Research. International alliance for responsible drinking. 2017 http://www.iard.org/wp-content/uploads/2016/01/IARD-General-Principles-for-Research.pdf

[37] O’Reilly D The Dublin principles. Email. February 5, 2000. British American Tobacco. Bates no. 325304888. 2000 http://legacy.library.ucsf.edu/tid/krj82a99/pdf?search=%22theinternationalcenterforalcoholpolicies%22.

[38] McCambridge J, Mialon M, Hawkins B Alcohol industry involvement in policy making: a systematic review. Addiction. In Press 2018 10.1111/add.14216 PMC6100095 29542202

[39] Fagerström KO, Bridgman K Tobacco harm reduction: the need for new products that can compete with cigarettes. Addict Behav 2014;39:507–11. 10.1016/j.addbeh.2013.11.002 24290207

[40] Human D Tobacco Tactics. 2018 http://www.tobaccotactics.org/index.php/Delon_Human (accessed 2nd Mar 2018).

[41] Sci-com. African science, society & policy indaba 2017. 2017 https://www.sci-com.eu/main/index.php/events/upcoming-events/144-african-science-society-policy-indaba-2017

[42] Hawkins B, McCambridge J Industry actors, think tanks, and alcohol policy in the United Kingdom. Am J Public Health 2014;104:1363–9. 10.2105/AJPH.2013.301858 24922137 PMC4103247

[43] Ulucanlar S, Fooks GJ, Gilmore AB The policy dystopia model: an interpretive analysis of tobacco industry political activity. PLoS Med 2016;13:e1002125 10.1371/journal.pmed.1002125 27649386 PMC5029800

[44] Lyness SM, McCambridge J The alcohol industry, charities and policy influence in the UK. Eur J Public Health 2014;24:557–61. 10.1093/eurpub/cku076 24913316 PMC4110957

[45] Andréasson S, McCambridge J Alcohol researchers should not accept funding from the alcohol industry: perspectives from brief interventions research. J Stud Alcohol Drugs 2016;77:537–40. 10.15288/jsad.2016.77.537 27340951

[46] Freudenberg N Public health advocacy to change corporate practices: implications for health education practice and research. Health Educ Behav 2005;32:298–319. discussion 55-62 10.1177/1090198105275044 15851541

[47] The Wellcome Trust. Wellcome statement: corporate event hire cancellation. 2017 https://wellcome.ac.uk/press-release/wellcome-statement-corporate-event-hire-cancellation

[48] Yach D Foundation for a smoke-free world. Lancet 2017;390:1807–10. 10.1016/S0140-6736(17)32602-8 29047446

[49] Malone RE, Chapman S, Gupta PC, et al A "Frank Statement" for the 21st Century? Tob Control 2017;26:611–2. 10.1136/tobaccocontrol-2017-054080 29066592

[50] World Health Organization. WHO statement on philip morris funded foundation for a smoke-free world. 2017 http://www.who.int/mediacentre/news/statements/2017/philip-morris-foundation/en/

[51] Daube M, Moodie R, McKee M Towards a smoke-free world? Philip Morris International’s new Foundation is not credible. Lancet 2017;390:1722–4. 10.1016/S0140-6736(17)32561-8 29047432

[52] Bero L Implications of the tobacco industry documents for public health and policy. Annu Rev Public Health 2003;24:267–88. 10.1146/annurev.publhealth.24.100901.140813 12415145

[53] McCambridge J, Hawkins B, Holden C Industry use of evidence to influence alcohol policy: a case study of submissions to the 2008 Scottish government consultation. PLoS Med 2013;10:e1001431 10.1371/journal.pmed.1001431 23630458 PMC3635861

[54] Collin J, Hill SE, Smith KE Merging alcohol giants threaten global health. BMJ 2015;351:h6087 10.1136/bmj.h6087 26578535

[55] Jernigan DH, Babor TF The concentration of the global alcohol industry and its penetration in the African region. Addiction 2015;110:551–60. 10.1111/add.12468 25771689

[56] Babor TF, Robaina K, Jernigan D The influence of industry actions on the availability of alcoholic beverages in the African region. Addiction 2015;110:561–71. 10.1111/add.12832 25510339

